# Partial erosion on under-methylated regions and chromatin reprogramming contribute to oncogene activation in *IDH* mutant gliomas

**DOI:** 10.1186/s13072-023-00490-x

**Published:** 2023-04-28

**Authors:** Xinyu Wang, Lijun Dai, Yang Liu, Chenghao Li, Dandan Fan, Yue Zhou, Pengcheng Li, Qingran Kong, Jianzhong Su

**Affiliations:** 1 School of Biomedical Engineering, School of Ophthalmology and Optometry and Eye Hospital, Wenzhou Medical University, Wenzhou, 325011 China; 2 Oujiang Laboratory, Zhejiang Lab for Regenerative Medicine, Vision and Brain Health, Wenzhou, 325011 Zhejiang China; 3 Wenzhou Institute, University of Chinese Academy of Sciences, Wenzhou, 325011 China

**Keywords:** DNA methylation, Chromatin, Oncogene, *IDH* mutation, Glioma

## Abstract

**Background:**

*IDH1/2* hotspot mutations are well known to drive oncogenic mutations in gliomas and are well-defined in the WHO 2021 classification of central nervous system tumors. Specifically, *IDH* mutations lead to aberrant hypermethylation of under-methylated regions (UMRs) in normal tissues through the disruption of TET enzymes. However, the chromatin reprogramming and transcriptional changes induced by *IDH*-related hypermethylation in gliomas remain unclear.

**Results:**

Here, we have developed a precise computational framework based on Hidden Markov Model to identify altered methylation states of UMRs at single-base resolution. By applying this framework to whole-genome bisulfite sequencing data from 75 normal brain tissues and 15 *IDH* mutant glioma tissues, we identified two distinct types of hypermethylated UMRs in *IDH* mutant gliomas. We named them partially hypermethylated UMRs (phUMRs) and fully hypermethylated UMRs (fhUMRs), respectively. We found that the phUMRs and fhUMRs exhibit distinct genomic features and chromatin states. Genes related to fhUMRs were more likely to be repressed in *IDH* mutant gliomas. In contrast, genes related to phUMRs were prone to be up-regulated in *IDH* mutant gliomas. Such activation of phUMR genes is associated with the accumulation of active H3K4me3 and the loss of H3K27me3, as well as H3K36me3 accumulation in gene bodies to maintain gene expression stability. In summary, partial erosion on UMRs was accompanied by locus-specific changes in key chromatin marks, which may contribute to oncogene activation.

**Conclusions:**

Our study provides a computational strategy for precise decoding of methylation encroachment patterns in *IDH* mutant gliomas, revealing potential mechanistic insights into chromatin reprogramming that contribute to oncogenesis.

**Supplementary Information:**

The online version contains supplementary material available at 10.1186/s13072-023-00490-x.

## Background

Hotspot mutations in the *IDH1* and *IDH2* genes are commonly found in malignant gliomas, acute myeloid leukemia, and various other cancers [1–3]. Several studies have suggested that these *IDH* hotspot mutations serve as early driver events in gliomagenesis, particularly in the development of diffuse low-grade gliomas and grade 4 astrocytomas [4–7]. Mutated *IDH* genes not only produce α-ketoglutarate (αKG), but also produce D-2-hydroxyglutarate (D2HG), which competitively inhibits iron-dependent hydroxylases, including TET family enzymes that mediate active DNA demethylation [8, 9]. Consequently, abnormal hypermethylation patterns of under-methylated regions have been discovered in *IDH* mutant gliomas, such as the glioma-CpG island methylator phenotype (G-CIMP) [10, 11].

Complex mechanisms underlying the interplay between DNA methylation and histone modification have been widely studied. Previous studies have revealed that H3K4 methylation could prevent de novo DNA methylation by disrupting the ADD domains of DNMT3A/B [12, 13]. DNMT3B could be recruited to gene bodies by H3K36me3 in actively transcribed genes [14], while DNMT3A could be recruited to shape the intergenic DNA methylation landscape by H3K36me2 [15]. Hence, the patients with DNMT3A PWWP domain mutations or NSD1 (H3K36me2 methyltransferase) mutations shares similar clinical features [16, 17]. In addition, the relationship between CpG methylation and H3K27me3 established by polycomb repressive complex 2 (PRC2) is complex and frequently altered in multiple cancer types [18, 19]. As the key drivers of oncogenesis, genetic variation of chromatin regulators lead to aberrant histone–DNA methylation crosstalk and promote cancer initiation and progression [20].

Although previous studies have demonstrated that *IDH* mutation is sufficient to establish aberrant hypermethylation in glioma [11, 21], the role of these altered methylation states and their relationship with chromatin features in gliomagenesis are not fully understood yet. Limited by the probe design bias of methylation microarray, previous studies of aberrant hypermethylation in *IDH* mutant gliomas mainly focused on CpG islands or promoters [6, 10, 22, 23]. These analyses were based on mean methylation level of all CpG sites for each genomic region, therefore, could not provide accurate quantification when methylation alteration only occurs at CpG islands or promoters partially [24, 25]. To comprehensively decipher the chromatin reprogramming and functional effect of these altered methylation states, it is required to precisely quantify the methylation changes at single CpG site resolution.

In this study, we developed a computational framework based on Hidden Markov Model to identify hypermethylated UMRs at single-base resolution in *IDH* mutant gliomas. The hypermethylated UMRs present a bimodal methylation status, named partially hypermethylated UMRs (phUMRs) and fully hypermethylated UMRs (fhUMRs). The phUMRs exhibit distinct genomic characteristics and histone signatures compared to fhUMRs. In contrast to the classical model that promoter methylation represses transcriptional activity, the genes within phUMRs on promoter were prone to be up-regulated. Up-regulated phUMR-related genes showed a marked increase in H3K4me3 and decrease in H3K27me3 signals, which may be associated with the inhibition of transcriptional repressors. Partial erosion on the promoter of oncogenes was linked to increased transcription accompanied by local changes of H3K4me3 and downstream changes of H3K36me3.

## Results

### Identification of hypermethylated UMRs in *IDH* mutant gliomas

**Fig. 1:**
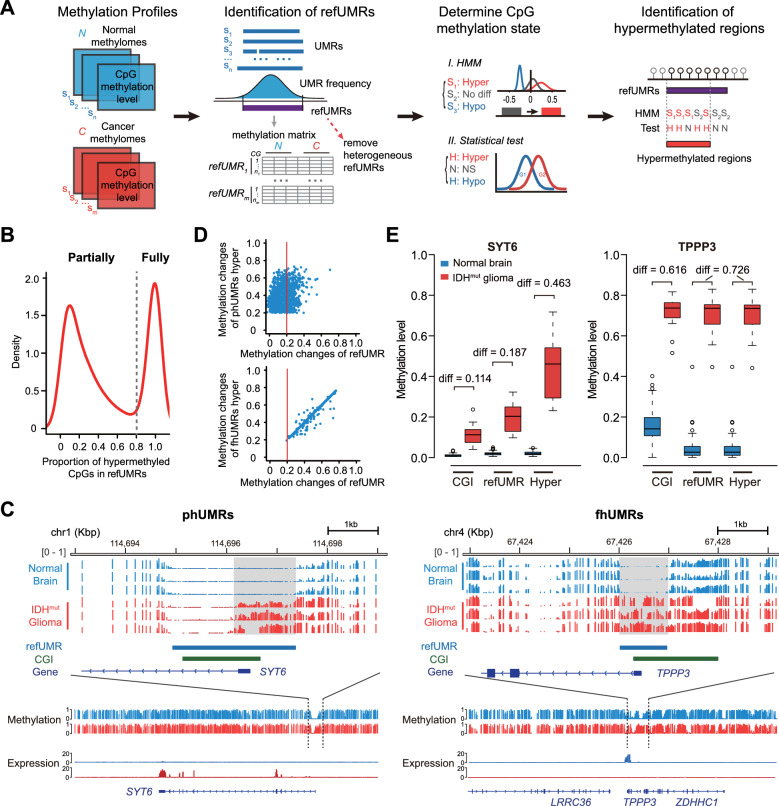
Identification of partially hypermethylated UMRs. **A** The computational framework for identification of hypermethylated regions from normal and cancer methylomes. **B** Bimodal distribution of the proportion of hypermethylated CpGs sites on under-methylated regions in *IDH*-mutant glioma. **C** Genome browser visualization of representative phUMR (*SYT6*) and fhUMR (*TPPP3*) in *IDH*-mutant gliomas. refUMR represents consistently under-methylated region in multiple brain tissues. Bottom panel represents methylation level track in a larger genome scale and the corresponding expression signals. **D** Comparison of methylation changes calculated using the hypermethylated regions (*y* axis) versus the entire reference under-methylated regions (*x* axis). Methylation change of each region was the absolute difference of mean methylation level for all CpG sites between *IDH*-mutant gliomas and normal brain samples. The frequently used methylation difference threshold 0.2 is denoted as the red line. **E** Comparison of methylation levels of SYT6 and TPPP3 using different benchmarks (*n* = 75 for normal brain tissues and *n* = 15 for *IDH* mutant glioma tissues). Hyper, hypermethylated regions in phUMRs or fhUMRs

To identify hypermethylated regions at base resolution between normal tissue and cancer tissue samples from WGBS data, we designed a computing framework based on Hidden Markov Model (Fig. 1A and “Methods” section). It consists of two core steps: (i) to identify consistently under-methylated regions (reference UMRs, refUMRs) in normal tissues. (ii) To identify internally hypermethylated region on reference UMRs in cancer tissues. The hypermethylation border were integrated from the hypermethylated CpG status determined by Hidden Markov Model and statistical test.

We applied this framework to publicly available WGBS datasets, including 72 normal brain tissues and 15 *IDH*-mutant gliomas [5, 26]. The internal consistency of WGBS data from different sources suggested that they could be incorporated to improve sample scale (Additional file 1: Fig. S1A, Additional file 2: Table S1). A total of 21,716 reference UMRs were identified in normal brain tissues, of which 2831 reference UMRs were abnormal hypermethylated in *IDH* mutant gliomas. To explore aberrant methylation changes within these hypermethylated UMRs, we examined the proportion of hypermethylated CpGs sites on under-methylated regions. Interestingly, not all these hypermethylated UMRs were fully hypermethylated, instead, a bimodal pattern was observed (Fig. 1B,C). This motivated us to divide abnormal hypermethylated regions into two categories: (i) partially hypermethylated UMRs (phUMRs), where CpG hypermethylation only occurs on a part of under-methylated regions, such as the methylation status of synaptotagmin *SYT6* promoter. (ii) Fully hypermethylated UMRs (fhUMRs), where hypermethylation occurs on the whole under-methylated regions, such as the methylation status of *TPPP3* promoter, a cerebrospinal fluid leak-related gene (Fig. 1C).

The traditional quantification method for methylation changes in *IDH* mutant gliomas involves calculating the average methylation level of all CG sites in CpG islands [6, 10]. However, compared to CpG islands, using reference UMRs as a benchmark could more accurately reflect the under-methylated regions in normal brain tissues. We identified 6495 novel UMRs that could not be identified as CpG islands. For UMRs that overlap with CpG islands, HMM approach provides a distinct advantage in detecting hypermethylated CpGs on UMRs (Additional file 1: Fig. S1B). Furthermore, quantifying methylation differences within hypermethylated regions using the average methylation level yields more accurate results than calculating the average across all CG sites within the UMRs. We found that phUMRs were more susceptible to the traditional mean methylation level method, with approximately 69.3% missing (Fig. 1D, Additional file 1: Fig. S1C). And the phUMRs and fhUMRs could provide a more comprehensive overview of aberrant methylation in *IDH* mutant gliomas compared to G-CIMP (Additional file 1: Fig. S1E–G). For instance, when using the SYT6 promoter CpG island as the reference region to quantify methylation changes, the quantitative methylation difference did not meet the usual threshold 0.2 for absolute methylation difference (Fig. 1E). These results suggested that our computational framework could provide a more accurate definition of aberrant hypermethylated regions in *IDH* mutant gliomas.

### Partially hypermethylated UMRs exhibit specific characteristics of genomic context and chromatin modifications

**Fig. 2:**
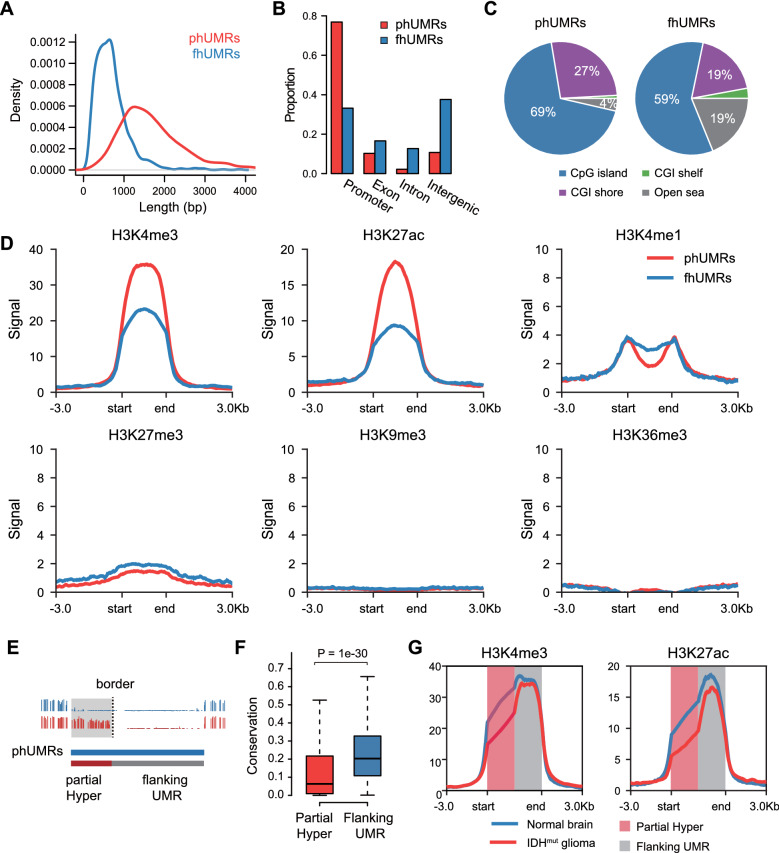
Features of partially and fully hypermethylated UMRs. **A** Length distribution of phUMRs and fhUMRs. **B** Genomic distribution of phUMRs and fhUMRs. **C** Percentage of phUMRs and fhUMRs overlap with CpG islands, CGI shores, CGI shelves and Open seas. **D** Average signal of histone modification for phUMRs and fhUMRs within ± 3 kb in normal brain tissue. **E** Diagram to show the definition of partial Hyper and flanking UMR in phUMRs. The border was determined by the hypermethylated CpGs among normal brain and *IDH* mutant glioma tissues. **F** Evolutionary conservation score of partial Hyper and flanking UMR. The *p*-value was tested using a two-tailed *t*-test. **G** Average signals of histone modification for phUMRs within ± 3 kb in normal brain and *IDH* mutant glioma tissues. Average ChIP-seq signals were scaled into 2 kb for partial Hyper and flanking UMRs, respectively

To characterize these two hypermethylated regions, we explored the genomic and chromatin features of phUMRs and fhUMRs. Compared with fhUMRs, phUMRs were longer and showed higher overlap with promoter and CpG islands. The fhUMRs were more represented as intergenic regions and CG-poor regions (Fig. 2A-C). Next, we combined the histone modification datasets of normal brain tissue to explore the histone characteristics of these two types of UMRs. It can be observed that phUMRs have stronger active histone modifications signals (H3K4me3, H3K27ac) than fhUMRs (Fig. 2D). The repressive histone modification H3K27me3 and the heterochromatin modification H3K9me3 were deficient in normal brain tissues. These results suggested that although both of phUMRs and fhUMRs possess low methylation level in normal brain tissues, they show different genomic context and chromatin characteristics.

In order to further investigate the internal context characteristics of the partial erosion on UMRs, we segmented phUMRs into partial Hyper and flanking UMRs according to the border of hypermethylated CpGs in *IDH* mutant gliomas (Fig. 2E). The evolutionary conservation of partial Hyper was lower than the flanking UMRs, implying that they occurred later during evolution and may have distinct regulatory functions in transcriptional switches (Fig. 2F). Compared to normal brain, partially hypermethylated regions in the matched *IDH* mutant gliomas samples were enriched by lower signals of the two active histone modifications, H3K4me3 and H3K27ac, while no significant difference could be observed in flanking under-methylated regions (Fig. 2G, Additional file 1: Fig. S3A).

The quantification of methylation levels in phUMR reflects the average methylation level of bulk tissues. To investigate the methylation status of phUMRs at the single-cell level, we analyzed an additional scRRBS dataset from *IDH* mutant gliomas [27]. We compared the methylation levels of partial Hyper and their flanking UMR in individual cells. Our analysis revealed that the partial Hyper in individual cells exhibited higher methylation levels compared to the flanking UMR (Additional file 1: Fig. S3C).

In addition, the phUMR-related genes were strongly enriched in neural development and cell differentiation in contrast to the fhUMR-related genes (Additional file 1: Fig. S4), suggesting the key regulatory roles of partially hypermethylated regions in cell fate determination. These results implied that although partially hypermethylated regions are linearly located close to their flanking under-methylated regions, their CpG methylation status may be shaped or maintained by different mechanisms, resulting in discrete methylation state transition among cell fate transitions.

### Promoter phUMR genes were prone to be overexpressed and involved in cancer pathway

**Fig. 3:**
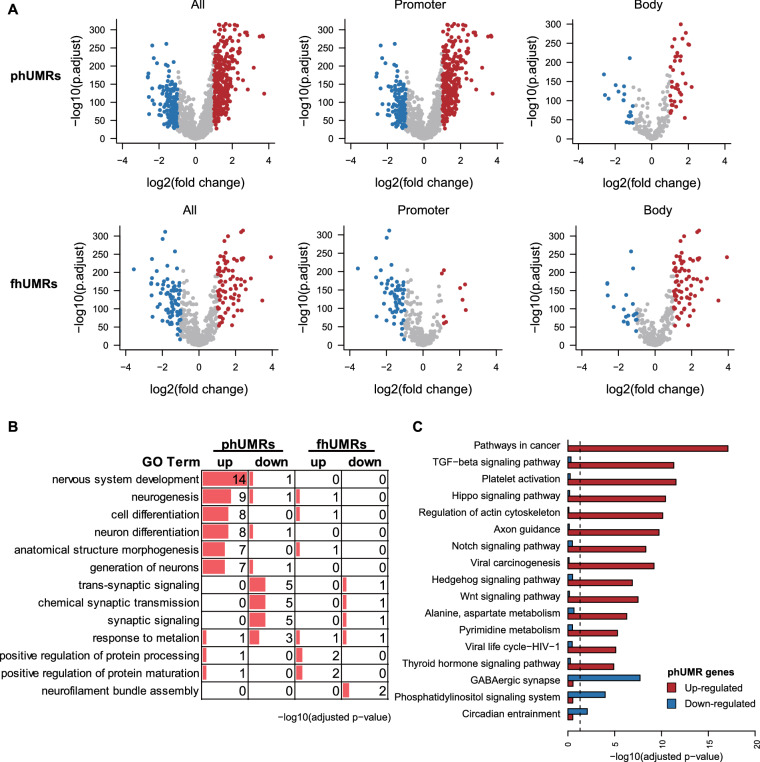
Transcriptional patterns of phUMR and fhUMR related genes. **A** Volcano plot displays the statistical significance (*y* axis) and fold change (*x* axis) of phUMR and fhUMR related genes between *IDH* mutant glioma and normal brain tissues (blue, down-regulated phUMR/fhUMR related genes; red, up-regulated phUMRs/fhUMRs related genes). **B** The functional annotation of up-regulated and down-regulated genes within phUMR/fhUMR on promoter. The *p*-values were adjusted using BH method. **C** The barplot shows the statistical significance (*y* axis) of KEGG terms for phUMR and fhUMR genes. The line indicates the adjusted *p*-value threshold 0.05

Previous studies have demonstrated that DNA methylation within the promoter or gene body shows varying effects on gene transcription [28, 29]. To explore the impact of DNA hypermethylation at specific genomic locations on the transcriptional regulation of adjacent genes, we mapped phUMRs and fhUMRs to genes based on their chromosomal positions. We then analyzed the transcriptional patterns of genes associated with phUMRs and fhUMRs across 1146 normal brain tissue samples from GTEx and 427 *IDH* mutant glioma samples from TCGA. Our analysis revealed that genes within fhUMR on promoter tended to be transcriptionally down-regulated in *IDH* mutant gliomas when compared to normal brain tissues (Fig. 3A). This finding is consistent with the classical DNA methylation regulatory model, which suggests that promoter hypermethylation inhibits gene transcription. And genes within phUMR or fhUMR on gene body tended to be up-regulated, which is consistent with previous research findings [30, 31]. In contrast, genes within phUMR on promoter were prone to be up-regulated (Fig. 3A). A similar trend was observed in a smaller cohort from DKFZ (Additional file 1: Fig. S5A, B). These results suggested that the transcriptional regulation pattern of phUMRs on promoter is distinct from the classical regulation mode of fhUMRs.

To further investigate the cellular diversity of phUMR and fhUMR-related aberrant transcriptional genes, we performed cell type enrichment analysis using a scRNA-seq dataset from *IDH*-mutant glioma [32]. Our analysis revealed that up-regulated genes related to phUMR exhibited relatively higher expression levels in microglia/macrophage cells, whereas down-regulated genes related to phUMR were enriched in oligodendrocytes (Additional file 1: Fig. S6). These results imply that up-regulated and down-regulated phUMR genes effect distinct cell types in bulk tissues of *IDH* mutant glioma.

We next explored the biological function of genes related to phUMRs and fhUMRs. For genes within phUMR on promoter, the up-regulated ones in *IDH* mutant gliomas were highly enriched in nervous system development and cell differentiation, while the down-regulated ones were enriched in synaptic signaling and chemical synaptic transmission (Fig. 3B). Additionally, genes within phUMR on gene body were related to negative regulation of biosynthetic process (Additional file 1: Fig. S5C). Pathway analysis indicated that the up-regulated genes within phUMR on promoter were significantly enriched in multiple cancer-related pathways, such as TGF-beta, Hippo, Notch and Wnt signaling pathways (Fig. 3C). Moreover, the down-regulated genes within phUMR on promoter were enriched in the GABAergic synapse pathway. Additionally, the down-regulated within fhUMR on promoter were enriched in the neuroactive ligand–receptor interaction pathway (Additional file 1: Fig. S5D). These results are consistent with previous studies indicating that D2HG produced by *IDH* mutation can block normal neural differentiation processes [3, 33, 34]. The distinct enrichment of genes associated with phUMRs and fhUMRs suggested that the up-regulated genes within phUMR on promoter may play key regulatory roles in gliomagenesis.

### The transcriptional programs of phUMR genes were related to interplay with DNA methylation and key chromatin modifications

**Fig. 4:**
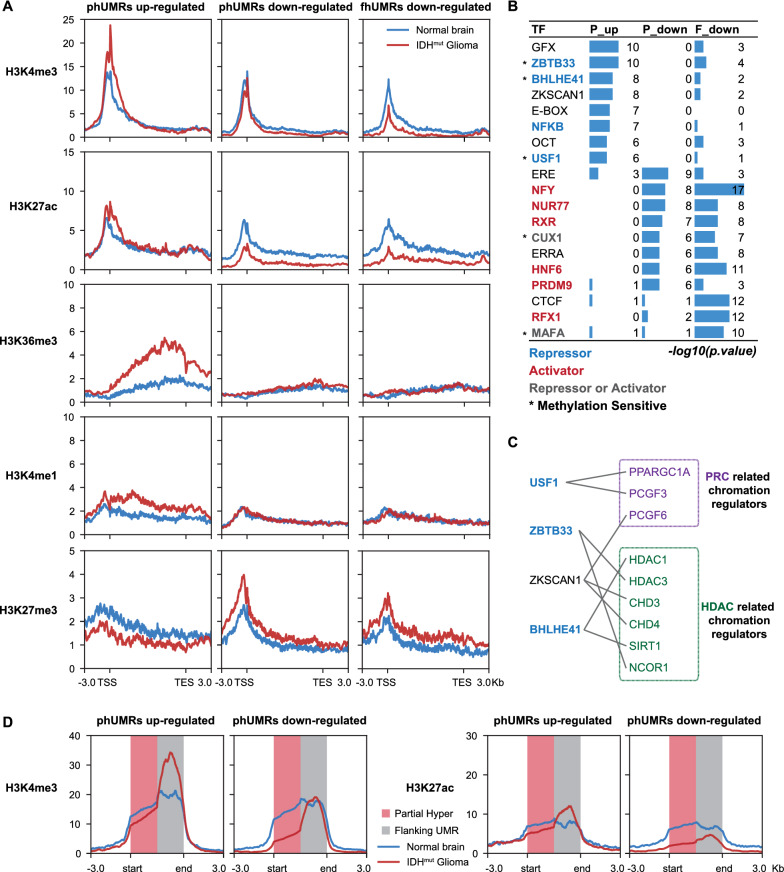
Differentially expressed phUMR and fhUMR related genes possess different chromatin features. **A** Average signals of histone modification mark around TSS and TES of differentially expressed genes between *IDH* mutant glioma and normal brain tissues. **B** Motif enrichment of these three hypermethylated regions with different transcriptional programs. P_up, hypermethylated regions in up-regulated phUMRs. P_down, hypermethylated regions in down-regulated phUMRs. F_down, hypermethylated regions of down-regulated fhUMRs. The color of transcription factors was apportioned by the effect to gene regulation. An asterisk indicates the transcription factor was previously reported to be methylation-sensitive. **C** The interaction of up-regulated phUMR-related transcription factors and PRC or HDAC related chromatin regulators. **D** Average signals of H3K4me3 and H3K27ac on partial Hyper and flanking UMR in up/down-regulated phUMRs. Average ChIP-seq signals were scaled into 2 kb for partial Hyper and flanking UMRs, respectively

Previous studies have reported the critical effects of the crosstalk between DNA methylation and histone modifications on transcriptional regulation [20]. To characterize the transcriptional programs of phUMRs more accurately, we analyzed the matched WGBS, RNA-seq and ChIP-seq datasets derived from a normal brain tissue sample (149 from Roadmap) and an *IDH* mutant glioma tissue sample (AK076 from DKFZ). A total of 338 differentially expressed genes within phUMR and 158 differentially expressed genes within fhUMR on promoter were identified. Up-regulated and down-regulated related phUMRs exhibit low methylation level on TSS and partial erosion in *IDH* mutant glioma. In contrast, hypermethylation of down-regulated related fhUMRs covered TSS (Additional file 1: Fig. S7). The up-regulated genes within phUMR on promoter possessed increased signals of active histone modifications (H3K4me3, H3K27ac, H3K4me1) and decreased signals of repressive histone modification H3K27me3 (Fig. 4A, Additional file 1: Fig. S8). In contrast, the down-regulated genes within phUMR or fhUMR showed decreased active histone modification signals and increased H3K27me3 signals. Notably, H3K36me3 was highly increased in the gene body of up-regulated genes within phUMR, which was consistent with the dependence of active transcription and H3K36me3 marks on gene body [14]. These results indicated that different transcriptional programs of hypermethylated gene may possess distinct interplay patterns of DNA methylation and chromatin modifications.

DNA methylation has been reported to affect the binding of transcription factors to DNA, thereby regulating downstream gene expression [35–37]. To further explore whether the different transcription regulatory patterns of phUMR on promoter are related to distinct transcription factors, we performed motif enrichment analysis on the up-regulated and down-regulated related phUMRs, respectively. We found that up-regulated related phUMRs tended to enrich methylation-sensitive repressor motifs, which binding to DNA could be disrupted by DNA methylation. In contrast, the down-regulated related phUMRs and fhUMRs tended to enrich transcriptional activator motifs (Fig. 4B). Previous studies reported that the interaction between transcription factors and chromatin regulators could shape chromatin state and regulate downstream gene transcription [36, 38]. Combined with high-confidence interaction pairs from BIOGRID, these repressors may interact with Polycomb (PRC) or histone deacetylase (HDAC) related chromatin regulators (Fig. 4C). These results suggested that the transcriptional up-regulation of phUMRs on promoter may be caused in part by inhibiting methylation-sensitive repressors, consistent with in some previous low-throughput experimental analyses [36, 39, 40].

To further explore the effect of the up- and down-regulated related phUMRs, we examined local changes of histone modifications for partial Hyper and their flanking UMRs (Fig. 2E). Although down-regulated and up-regulated phUMRs showed consistent local methylation changes, but they exhibited different patterns of changes in chromatin signatures (Fig. 4D, Additional file 1: Fig. S9A). For up-regulated related phUMRs on promoter, increased active modification signals were located at the flanking UMRs, but not at partial Hyper regions (Fig. 4D, Additional file 1: Fig. S9A). However, the down-regulated related phUMRs on promoter showed a decreased tendency of active modification signals at partial Hyper regions, which may be caused by the well-documented antagonism of H3K4me3 and de novo methylation by DNMT3A/B [20]. Similar phenomena to these results were obtained on matched methylation, histone modification, and transcription data in an additional pair of samples (150 from Roadmap vs. AK213 from DKFZ, Additional file 1: Figs. S8, S9). These results indicated that differential transcriptional outcome of promoter partial methylation erosion may be related to distinct cooperative regulatory modes between DNA methylation and histone modifications.

### Oncogene activation accompanied by partial methylation erosion and local-specific changes of chromatin mark

**Fig. 5:**
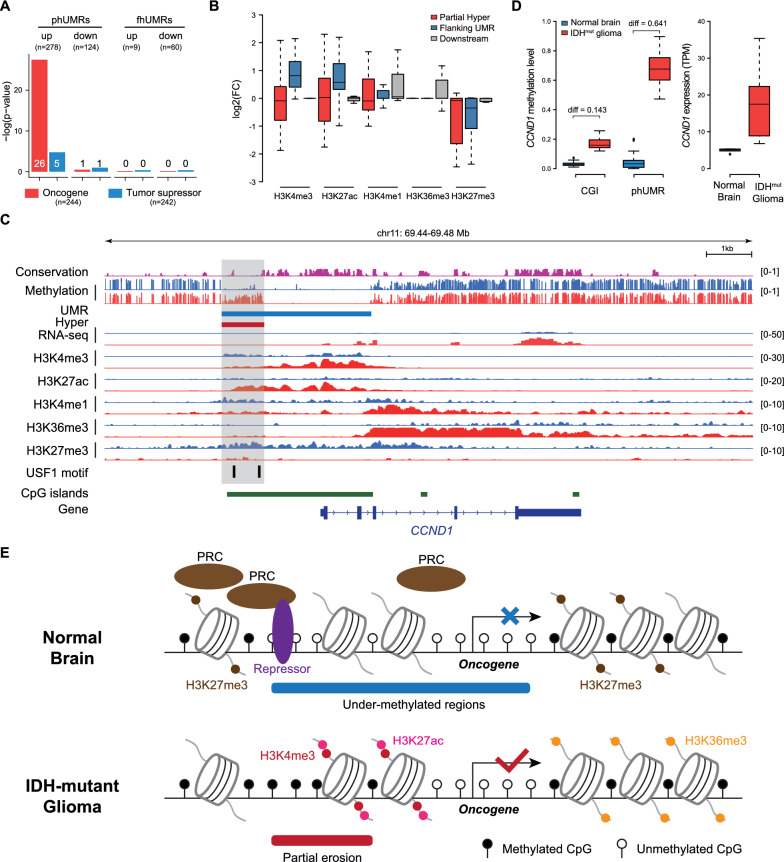
Partially hypermethylated UMRs were accompanied by local changes of key histone modifications contributed to oncogene activation. **A** The barplot displays the overlap between up-regulated phUMR-related genes and oncogenes. *p*-value was computed using Fisher’s exact test. The specific numbers of overlapping genes between phUMRs/fhUMRs related differentially expressed genes and cancer genes are displayed on the bars. **B** The Boxplot shows fold changes of average histone modification signals at partial Hyper, flanking UMR and downstream regions of up-regulated oncogenes. Partial Hyper and flanking UMR was determined by the border of hypermethylated CpGs in *IDH* mutant gliomas, and downstream represent the region from the border of phUMR to TES of oncogene. **C** Genome Browser tracks depict DNA methylation, gene transcription and histone modification changes across CCND1 in *IDH* mutant glioma (red) and normal brain tissue (blue). **D** Boxplot compared methylation level changes (left) or gene expression (right) of CCND1 among *IDH* mutant glioma and normal brain tissues. CGI, methylation level was quantified as mean methylation level of CpG sites in promoter CpG island of CCND1. phUMR: methylation level was quantified as mean methylation level of CpG sites in hypermethylated regions on phUMRs of CCND1. **E** Hypothesis model of oncogene activation accompanied by partial methylation erosion and chromatin reprogramming

Aberrant epigenetic changes could lead to abnormal transcription of cancer genes including oncogene activation and tumor suppressor silencing [41]. To explore the functional effect of phUMRs and fhUMRs in glioma initiation and progression, we investigated the roles of phUMRs and fhUMRs in oncogene activation and tumor suppressor inactivation. We found that the phUMR-related up-regulated genes were significantly enriched in oncogenes (Fig. 5A). For example, an obvious phUMR was observed at the promoter of *PDGFRA*, a well-known prominent glioma oncogene (Additional file 1: Fig. S10). *IDH* mutation was reported as the initiating event in glioma progression, which was thought to block neural differentiation [3]. The stemness genes activated by aberrant hypermethylation in *IDH* mutation gliomas may provide selective growth advantages to tumor cells and promote gliomagenesis.

We further investigated local changes of distinct histone modifications for these activated oncogenes within phUMR on promoter. These oncogenes were segmented into partial Hyper, flanking UMRs and downstream body (from the border of UMR to TES of oncogene) according to the border of methylation status. We found that methylation erosion of these up-regulated oncogenes within promoter phUMR were accompanied by local changes of H3K27me3, H3K4me3 and downstream changes of H3K36me3 (Fig. 5B, Additional file 1: Fig. S11). Additionally, loss of H3K27me3 were found in partial Hyper and flanking UMRs. For example, at the promoter of *CCND1*, a key gene for cell cycle progression, loss of broad H3K27me3 signal and increase of H3K4me3 signal in flanking UMRs were found. Furthermore, the gene body of *CCND1* presented increased H3K36me3 and H3K4me1 signals (Fig. 5C). Interestingly, the motif of methylation-sensitive repressor USF1 was located in the partial Hyper but not in the flanking UMR of *CCND1*. Previous studies reported that USF1 could interact with PCGF3, which is a subunit of the PRC complex. Additionally, the methylation change of promoter CGI for *CCND1* could not meet the methylation difference threshold of G-CIMP (Fig. 5D). These results suggested that oncogene activation may be caused by the disruption of the binding of methylation-sensitive repressors, leading to chromatin reprogramming of this region.

## Discussion

In this study, we developed a computational framework and defined two types of hypermethylated UMRs with distinct transcriptional programs. Due to the high cost of sequencing, most tumor methylation data were measured using 450K/850K microarrays, which possess large-scale samples and uniform CpG probes. The limitation is that the number of CG sites is incomplete, and information on CG density and surrounding context is lost. Therefore, traditionally identified aberrant hypermethylated regions in *IDH* mutant gliomas were based on the average changes of methylation, which ignores the surrounding sequence information. Compared to DNA methylation microarrays, WGBS data could provide a more comprehensive information overview, leading to the discovery of a series of distinct methylation alteration regions in cancer [42–44]. The distinct characteristics and functions of these DNA methylation alteration regions indicate that the regulatory function of DNA methylation could highly depend on the contextual information of genomic locations.

The phUMRs and fhUMRs were de novo identified based on under-methylated regions in control samples. Mathematically, "partial" may be caused by more than two discrete methylation alteration regions with inconsistent methylation changes at this chromosomal position under different physiological and pathological conditions. Although these adjacent methylation alteration regions are linearly close in chromosomal locations, their methylation states may be regulated by different regulators and possess different transcriptional regulatory functions. This is an effective enhancement of the classical DNA methylation regulatory model. Meanwhile, like other methylation alteration regions based on methylation level, the accuracy of phUMR also depends on the methylation datasets. Therefore, a uniform reference methylation region set could benefit the functional analysis of DNA methylation and identification of disease-related methylation markers in the future.

Through data mining of public WGBS data in *IDH* mutant gliomas, we observed different transcriptional regulation patterns in phUMRs and fhUMRs. These findings could provide important insights into the transcriptional regulation of DNA methylation. However, there is sparse data available on repressor ChIP-seq and matching WGBS data related to phUMRs affecting gene expression. To achieve a more precise interpretation of DNA methylation alterations in cancer, we need appropriate physiological and pathological models and generate more comprehensive matched multi-omics data. Moreover, CRISPR screening technology has been widely used in recent years to identify novel chromatin regulators [45–47], and could be employed to identify novel repressors on the phUMR that are related to the up-regulation of methylation transcription.

The crosstalk between DNA methylation and histone modifications determines the expression level of adjacent gene transcription. Our study revealed that partial methylation erosion and chromatin reprogramming were involved in oncogene activation in *IDH* mutant gliomas. Based on our study, we propose a model of oncogene activated by partial methylation erosion (Fig. 5E), which complement the classic model of promoter hypomethylation leading to oncogene activation. In normal brain tissues, oncogenes are silenced by repressive histone modifications such as H3K27me3, which may be recruited by methylation-sensitive transcriptional repressors. In *IDH* mutant gliomas, neoplastic hypermethylation of transcriptional repressor binding sites leads to the destruction of this binding, resulting in the accumulation of active histone modifications, and finally promote downstream oncogene activation. However, the sequence of aberrant epigenetic reprogramming events is not clear, so further studies may be able to use some representative oncogenes to validate molecular mechanism in the future.

Combined with the single-cell methylation and transcriptome data of *IDH*-mutant gliomas, we observed the internal differential methylation status of phUMR at the single-cell level, and up-regulated genes related to phUMR were enriched in microglia/macrophage cell types and malignant tumor cells. In the future, the application of single-cell multi-omics detection technologies such as scM&T-seq [48] and scTrio-seq [49] may provide more accurate regulatory function of phUMR on gene transcription at single-cell level.

It should be noted that *IDH* mutant gliomas were a special tumor type with defined driver events. Due to the high degree of intra-heterogeneity within other tumors, it is difficult to completely decipher the pan-cancer abnormal methylation pattern with limited tumor WGBS samples nowadays. More WGBS data resources will be needed in the future. Meanwhile, the application of targeted methylation editing [50, 51] will help to accurately understand the role of aberrant DNA methylation in transcriptional regulation and tumorigenesis.

## Conclusions

In summary, we developed a computational framework to accurately identify aberrant hypermethylation in *IDH* mutant gliomas. We defined two distinct subsets of DNA hypermethylation pattern regions (phUMRs/fhUMRs) and depicted their regulatory programs, respectively. In contrast to the classical model that promoter methylation represses transcriptional activity for fhUMR related genes, the genes within phUMRs on promoter were prone to be up-regulated and involved in cancer pathways. We further found that the phUMR contributes to oncogene activation by the interplay with key chromatin marks, which implies a novel model of oncogene activated by partial methylation erosion.

## Methods

### WGBS data sources

WGBS data of 75 normal brain tissue samples were obtained from Roadmap and GSE96615 [52, 53]. WGBS data of 15 *IDH* mutant gliomas were obtained from two cancer centers, TCGA and DKFZ [5, 26]. List of these WGBS samples used in this study is shown in Additional file 2: Table S1. For each CpG site, the reads on the positive and negative strands were merged together to improve read coverage. Only the CpG sites with more than five reads were considered for analysis, and the CpG sites on the scaffold were removed. Principal component analysis (PCA) analysis of CpG island methylation level was used to measure the consistency of WGBS samples from different sources.

### Identification of phUMRs and fhUMRs

For WGBS data of normal brain tissues and *IDH* mutant glioma tissues, UMRs were first identified for each WGBS sample in normal brain tissues, and then the frequency of UMR occurrence was integrated to identify consistent under-methylated regions in normal brain tissues (reference UMR). The detail algorithm was shown in our previous study [29].

Based on the reference UMRs identified in normal brain tissues, average methylation level of individual CpG site in the two groups was calculated separately. A three-state Hidden Markov Model was used to determine the differential methylation status: hypermethylated (Hyper), hypomethylated (Hypo), no difference (No diff). The emission probability matrix for these three states were modeled using Gaussian distribution, and the mean and variance were estimated using the methylation difference between WGBS data for *IDH* mutant gliomas and normal brain tissues. Transition probability was estimated by counting the methylation levels of adjacent CpG sites. For each refUMRs, the initial differential methylation state of the first CpG site was set by calculating the average methylation level for that region. RHmm (version 2.0.2) was used to assign differential status to each CpG site.

Methylation matrix of each CpG site for each refUMR was generated. The *p*-value for each CpG site within the reference UMRs was calculated using the two-tailed *t*-test. The difference status of CpG sites with an FDR-corrected *p*-value of less than 0.05 and an absolute methylation difference ≥ 0.2 was determined as Hyper.

The CpG sites identified as Hyper by Hidden Markov Model or statistics testing were merged into methylated regions. According to the bimodal distribution curve of the proportion of hypermethylated CpG sites in reference UMRs, the proportion of CpG sites located in the hypermethylated region is less than 0.8 and the number of CGs ≥ 5 was defined as phUMRs. And the proportion of CpG sites located in the hypermethylated regions is more than 0.8 was defined as fhUMRs.

This computational framework is publicly available at https://github.com/wangxinyush/IDH_phUMR.

### Curation of G-CIMP

The definition of G-CIMP was inspired by previous studies [10, 23], in which CpG islands with an FDR-corrected *p*-value less than 0.05 and an absolute methylation difference greater than 0.2 were defined as G-CIMP. Annotation information for CpG island regions was obtained from UCSC [54]. First, we calculated the mean methylation level of all detected CpG sites for 27,718 CpG islands in each WGBS sample. The CpG islands located in the scaffold were removed. Secondly, differentially methylated CpG islands were identified using *t*-test between normal brain and *IDH* mutant glioma tissues. The CpG islands that passed the statistical test threshold and methylation absolute difference threshold were identified as G-CIMP. To compare the quantitative methylation value for different methylation region (CGI, Hyper, refUMR), we used the detected CpGs in each region to calculate the mean methylation level.

### Annotation of methylated regions and conservation analysis

The 2 kb downstream of the CpG island was defined as CpG island shore, the extending 2 kb from the CpG island shore was defined as CpG island shelf, and the remaining genomic regions was defined as open sea. The genomic position annotation of methylated regions was used ChIPseeker v1.5.1 [55]. The comprehensive annotation file of genes was downloaded from Gencode v23 [56].

For conservation analysis, PhastCons scores for human genome at single-base resolution were downloaded from UCSC [54]. The evolutionary conservation score of a methylated region was calculated by the average value of the evolutionary conservation scores of all bases in the region.

### Functional enrichment analysis

The phUMR and fhUMR related genes were defined as 2 kb upstream of TSS to TES. GO functional annotation of a specific gene sets was performed using ClusterProfiler 4.4.4 [57], and the results of KEGG functional annotation were obtained from the Metascape online tool [58]. The genes marked by both phUMR and fhUMR were removed. The list of oncogenes and tumor suppressor genes was collected from COSMIC [59], the genes that were marked as both oncogenes and tumor suppressors were removed.

### ChIP-seq data processing and analysis

Six type of histone modification signals (H3K4me3, H3K4me1, H3K27ac, H3K36me3, H3K27me3, H3K9me3) matched with normal brain WGBS samples were obtained from Roadmap [52], and the histone modification signals matched with *IDH* mutant glioma WGBS samples were obtained from DKFZ [26]. To compare normal brain and *IDH* mutant glioma samples, ChIP-seq data from Roadmap were processed as the same analysis pipeline in DKFZ [26]. Signal values of histone modifications were calculated using the SES method and visualized using deepTools 3.3.0 [60].

### RNA-seq data processing and analysis

The gene expression data of GTEx and TCGA used in this study were obtained from UCSC Xena (https://xenabrowser.net/datapages/). The gene expression data of normal brain and *IDH* mutant glioma tissues were obtained from Roadmap and DKFZ [26]. For multiple groups of samples, FDR-corrected *t*-test was used for TPM values, and the fold change was required to be greater than or equal to 2. For paired samples, only the fold change was used as the threshold.

### Single-cell RRBS and RNA-seq data analysis

Single-cell RRBS data of 6 *IDH* mutant gliomas were obtained from the Johnson et al. study [27]. Sample SM004 was excluded from further analysis due to a low cell count (*n* = 21). For each cell, the methylation status of partial Hyper and their flanking UMRs were calculated by the mean methylation level of detected CpG sites.

Single-cell RNA-seq data of *IDH* mutant gliomas were obtained from GSE89567 [32]. The cell type and clustering coordinate information were obtained from Single Cell Portal (https://singlecell.broadinstitute.org/single_cell). Gene set activity of phUMR/fhUMR related genes was calculated by AUCell (v1.14.0) [61]. AUCell_buildRankings algorithm was used for ranking model building, and then AUCell_calcAUC method was used to calculate the "Area Under the Curve" (AUC) scores.

### Cross-sample ChIP-seq analysis of partial Hyper and flanking UMR

For each phUMR, the region was segmented into partial Hyper and flanking UMR according to the border of hypermethylated CpGs in *IDH* mutant gliomas. We computed ChIP-seq signals for these two types of regions, respectively, using deeptools 3.3.0 computeMatrix. Both partial Hyper and flanking UMR were scaled into 2 kb. For each matched partially Hyper and flanking UMR, the output signal per window was merged base on the border of these two types of regions. And the merged signal matrix (upstream, partial Hyper, flanking UMR, downstream) was visualized using deeptools 3.3.0 plotHeatmap.

### Motif enrichment analysis

Motif enrichment analysis of methylated regions was used Homer findMotifsGenome.pl script [62]. The methylated regions with reverse transcriptional regulation were used as the background. The methylation preferences of TF motifs with methylation-sensitive SELEX were obtained from the previous study [63]. The interaction pair of transcriptional factor and chromatin regulator was downloaded from BioGRID [64].

## Supplementary Information

**Additional file 1**. **Fig. S1**: The refUMRs provide a better definition of hypermethylated regions in *IDH* mutant gliomas. **Fig. S2**: Partial hyper possess distinct features compared with flanking UMRs. **Fig. S3**: Chromatin features of fhUMRs and phUMRs. **Fig. S4**: GO and KEGG functional annotation of phUMR and fhUMR related genes. **Fig. S5**: Transcriptional tendency of phUMR and fhUMR genes in DKFZ. **Fig. S6**: Cell type enrichment analysis of phUMRs related genes. **Fig. S7**: Heatmaps of methylation signals at up- and down-regulated phUMR/fhUMR genes. **Fig. S8**: Histone modification changes in differentially expressed genes within phUMR or fhUMR. **Fig. S9**: Local histone modification changes on partial Hyper and flanking UMR in phUMRs. **Fig. S10**: Partial methylation erosion on the promoter of glioma related genes. **Fig. S11**: Local changes of average histone modification signals at partial Hyper, flanking UMR and downstream regions of up-regulated oncogenes.

**Additional file 2**. **Table S1**: Sample information of WGBS data.

**Additional file 3**. **Table S2**: The genomic context of phUMRs and fhUMRs.

## Data Availability

All the codes used are publicly available at https://github.com/wangxinyush/IDH_phUMR.

## Abbreviations

UMRs: Under-methylated regions
phUMR: Partially hypermethylated UMRs
fhUMR: Fully hypermethylated UMRs
refUMR: Reference UMR
G-CIMP: Glioma-CpG island methylator phenotype
D2HG: D-2-Hydroxyglutarate
PRC2: Polycomb repressive complex 2
HDAC: Histone deacetylase
PCA: Principal component analysis
Hyper: Hypermethylated
CGI: CpG islands
