# Supplementary material for: Partial erosion on under-methylated regions and chromatin reprogramming contribute to oncogene activation in IDH mutant gliomas
Source: Epigenetics Chromatin. 2023 Apr 28;16:13. doi: 10.1186/s13072-023-00490-x (PMC10142198; doi:10.1186/s13072-023-00490-x)
Supplement: Supplementary file 1 — Additional file 1. Fig. S1: The refUMRs provide a better definition of hypermethylated regions in IDH mutant gliomas. Fig. S2: Partial hyper possess distinct features compared with flanking UMRs. Fig. S3: Chromatin features of fhUMRs and phUMRs. Fig. S4: GO and KEGG functional annotation of phUMR and fhUMR related genes. Fig. S5: Transcriptional tendency of phUMR and fhUMR genes in DKFZ. Fig. S6: Cell type enrichment analysis of phUMRs related genes. Fig. S7: Heatmaps of methylation signals at up- and down-regulated phUMR/fhUMR genes. Fig. S8: Histone modification changes in differentially expressed genes within phUMR or fhUMR. Fig. S9: Local histone modification changes on partial Hyper and flanking UMR in phUMRs. Fig. S10: Partial methylation erosion on the promoter of glioma related genes. Fig. S11: Local changes of average histone modification signals at partial Hyper, flanking UMR and downstream regions of up-regulated oncogenes. [file 13072_2023_490_MOESM1_ESM.docx]

**Additional file 1**


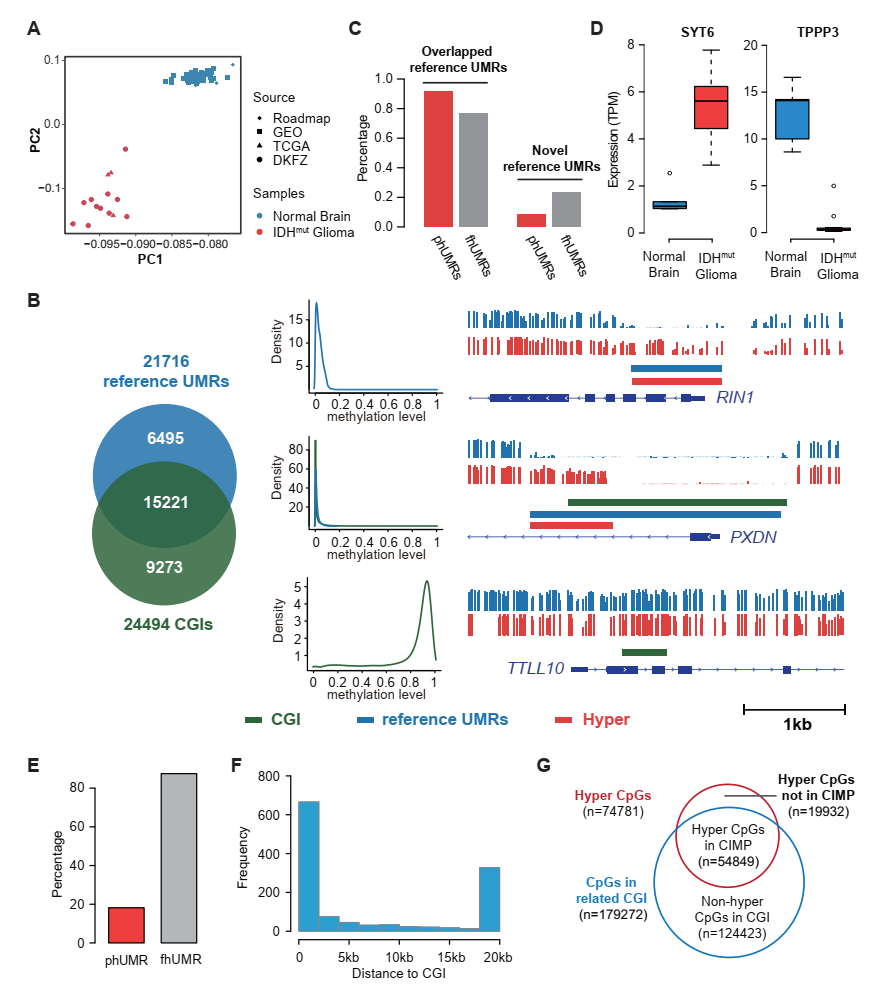


**Figure S1. The refUMRs provide a better definition of hypermethylated regions in *IDH* mutant gliomas.** A) PCA of distances based on average methylation levels of CpG islands in normal brain and *IDH* mutant glioma tissues. Each point is a sample, and samples from different datasets were shown as distinct point symbols (n = 75 for GSE96615, n =3 for Roadmap, n = 12 for DKFZ, and n = 3 for TCGA). B) The reference UMRs show more accurate boundaries than CpG islands in normal brain tissues. Left: Venn plot of reference UMRs and CGIs. Middle: distribution of methylation level in brain normal tissues (blue, average methylation of reference UMRs; green, average methylation of CGIs). A large of CGIs could not be identified as reference UMRs because of their high methylation level in normal brain tissues. Right: Track visualization of methylation on reference UMRs and CGIs in normal brain (blue) and *IDH* mutant glioma tissue (red). Hyper, hypermethylated regions at reference UMRs in *IDH* mutant gliomas. C) Proportion of phUMRs and fhUMRs identified in reference UMRs overlapped with CGIs and novel reference UMRs. D) Expression changes of *SYT6* and *TPPP3* among normal brain and IDH mutant glioma tissues. TPM, Transcripts Per Million. E) The bar plot displays the percentage of phUMRs/fhUMRs that could not be identified as CIMP. F) The distance to CpG islands for the phUMR/fhUMRs that did not overlap with CIMP. The bimodal distribution suggests that the hypermethylated CpGs are located within adjacent CGI regions (CGI shores) or distal regions (such as enhancers). G) CIMP could not reflect all hypermethylated CpGs, and most of the CpGs in CIMP are not hypermethylated, which can lead to inaccurate quantification.


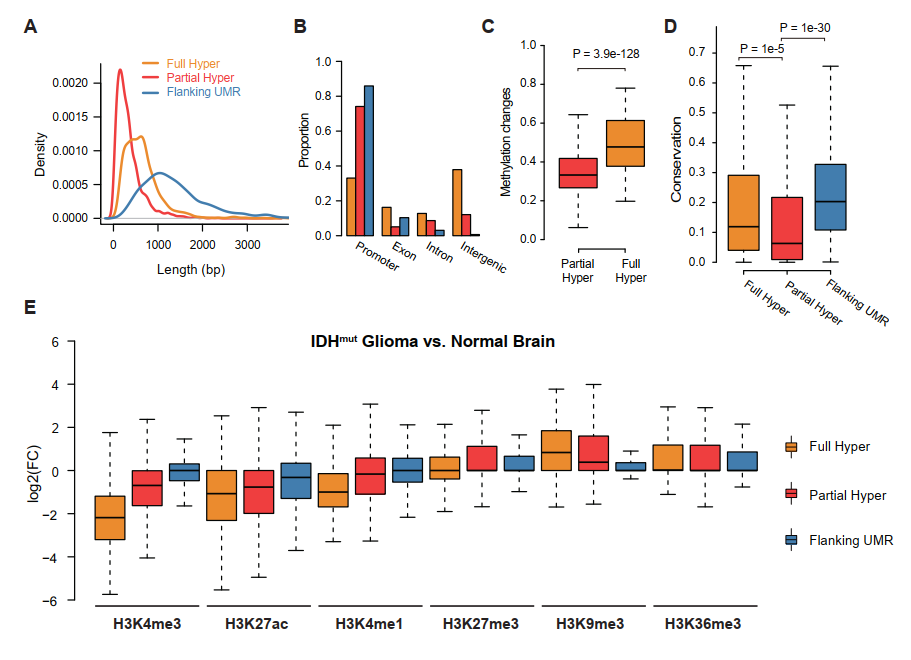


**Figure S2. partial Hyper possess distinct features compared with flanking UMRs.** A) Length distribution of full Hyper, partial hyper and flanking UMRs. Full Hyper represents hypermethylated regions in fhUMRs. B) Genomic distribution of full Hyper, partial Hyper and flanking UMRs. C) Methylation changes of partial Hyper and full Hyper. Methylation changes were calculated by absolute methylation level difference among *IDH* mutant glioma and normal brain tissues. P-values were tested using two-tailed t.test. D) Evolutionary conservation score of full Hyper, partial Hyper and flanking UMR. P-values were tested using a two-tailed t.test. E) Fold changes of average ChIP-seq signals for six histone modifications among *IDH* mutant glioma and normal brain tissues.


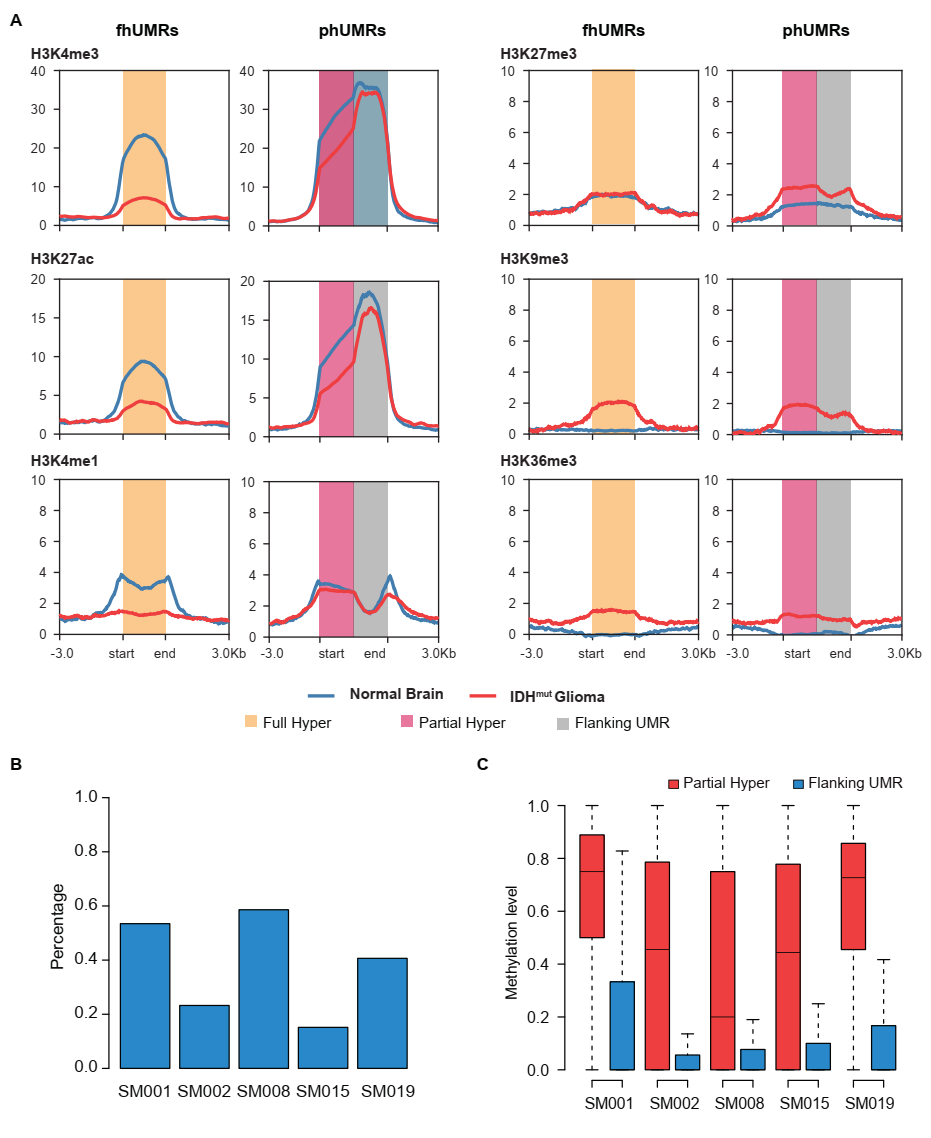


**Figure S3. Chromatin features of fhUMRs and phUMRs.** A) Average signals of histone modification on fhUMRs and phUMRs. PhUMRs were segmented into partial Hyper and flanking UMR based on methylation changes among *IDH* mutant glioma and normal brain tissues. Average ChIP-seq signals were scaled into 2kb for partial Hyper and flanking UMRs respectively. B) The barplot shows the percentage of detected phUMRs in merged scRRBS data. C) The methylation levels of partial Hyper and flanking UMR for each cell. Each point represents the mean methylation level of partial Hyper or flanking UMR in a single cell.


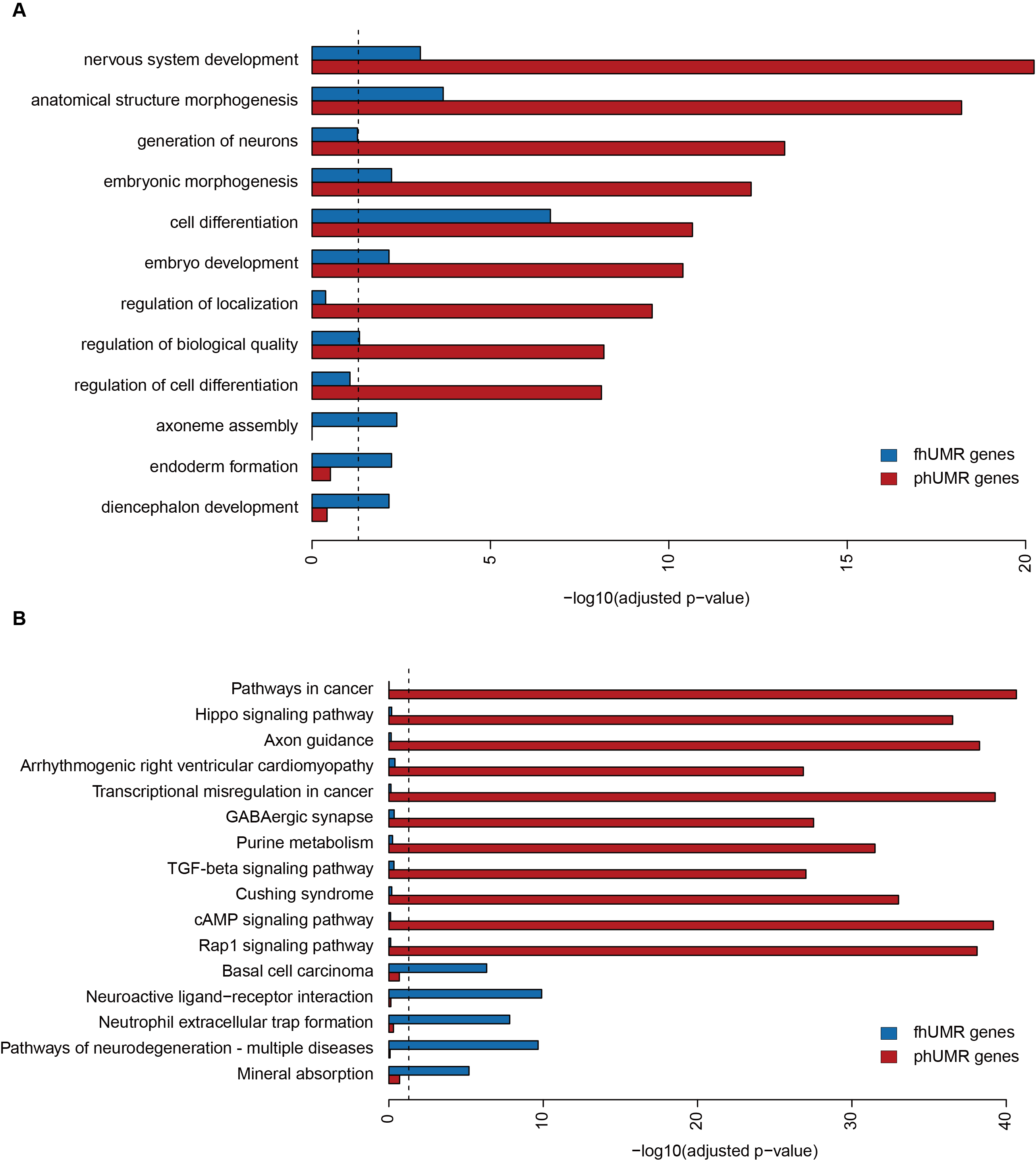


**Figure S4. GO and KEGG functional annotation of phUMR and fhUMR related genes.** The barplots shows the statistical significance (y axis) of GO (up) or KEGG (bottom) terms for phUMR and fhUMR genes. All the P-values were adjusted using BH method. The line indicates the adjusted p-value threshold 0.05.


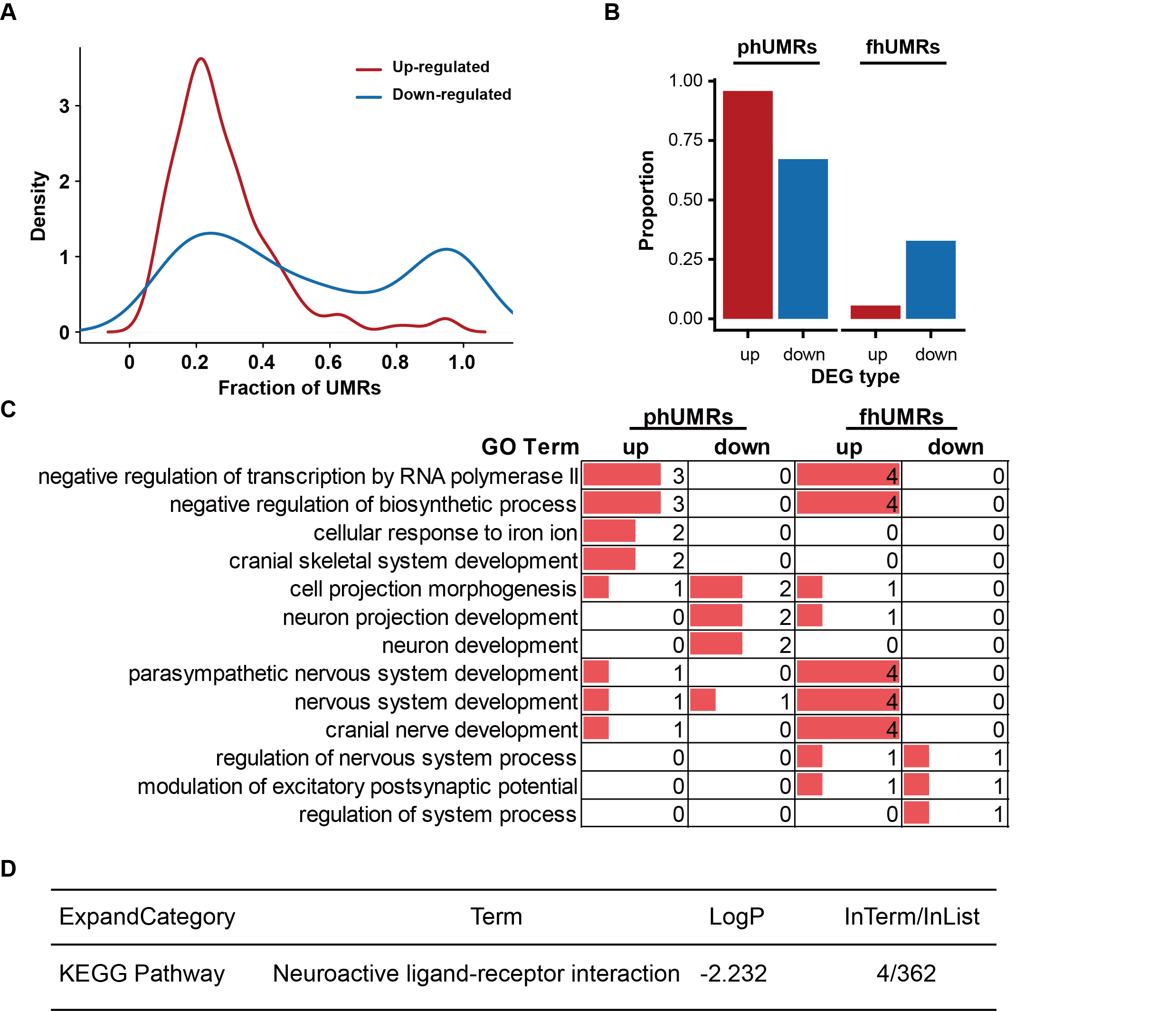


**Figure S5. Transcriptional tendency of phUMR and fhUMR genes in DKFZ.** A) Distribution of the fraction of hypermethylated regions in UMRs for up- and down-regulated genes in DKFZ. B) The boxplot shows phUMR related genes on promoter in DKFZ were prone to be up-regulated. C) GO functional annotation of up-regulated and down-regulated genes within phUMR/fhUMR on gene body. The p-values were adjusted using BH method. D) KEGG functional annotation of fhUMR related genes.


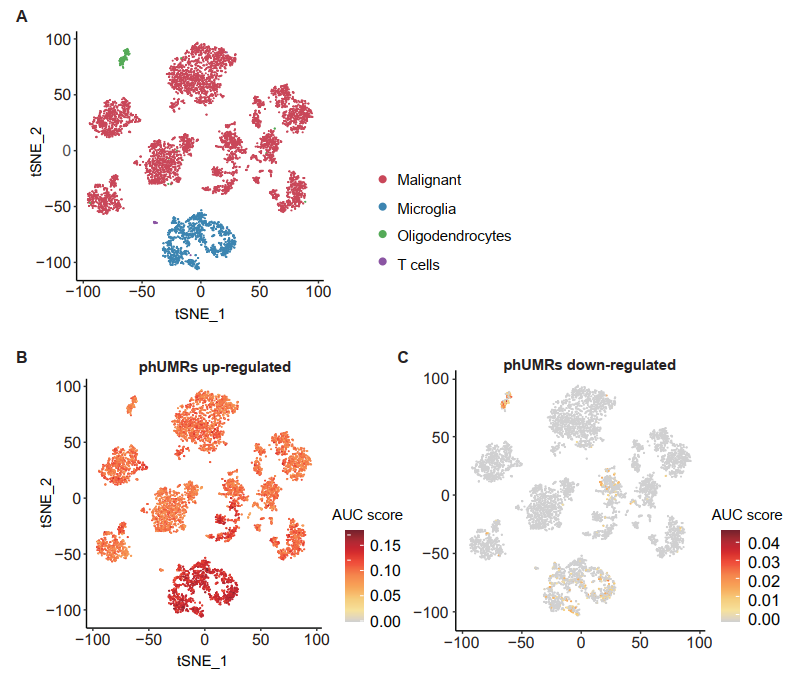


**Figure S6. Cell type enrichment analysis of phUMRs related genes.** A) Cell clusters of *IDH* mutant gliomas. The cell type and clustering coordinate information were obtained from the Single Cell Portal (https://singlecell.broadinstitute.org/single_cell). B) Gene set activity of up-regulated genes within phUMR. C) Gene set activity of down-regulated genes within phUMR. AUC cell score was used to estimate the proportion of genes in the gene-set that are highly expressed in each cell.


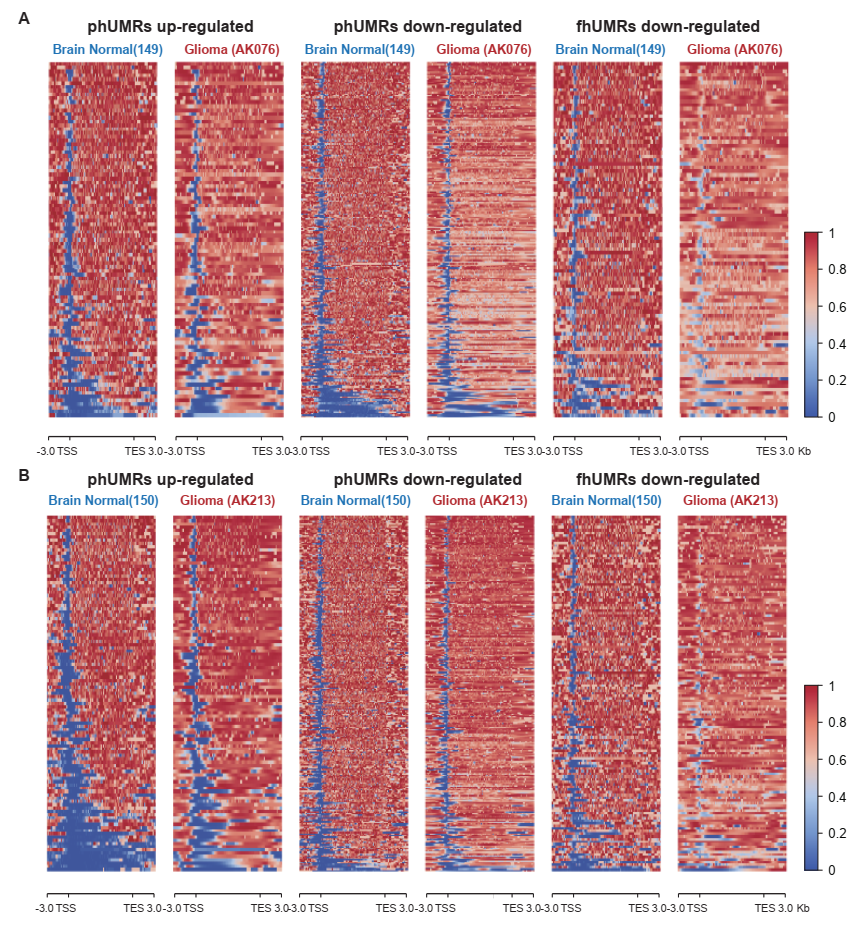


**Figure S7. Heatmaps of methylation signals at up- and down-regulated phUMR/fhUMR genes.** The gene lengths were scaled into 9kb for phUMR and fhUMR related up/down-regulated genes.


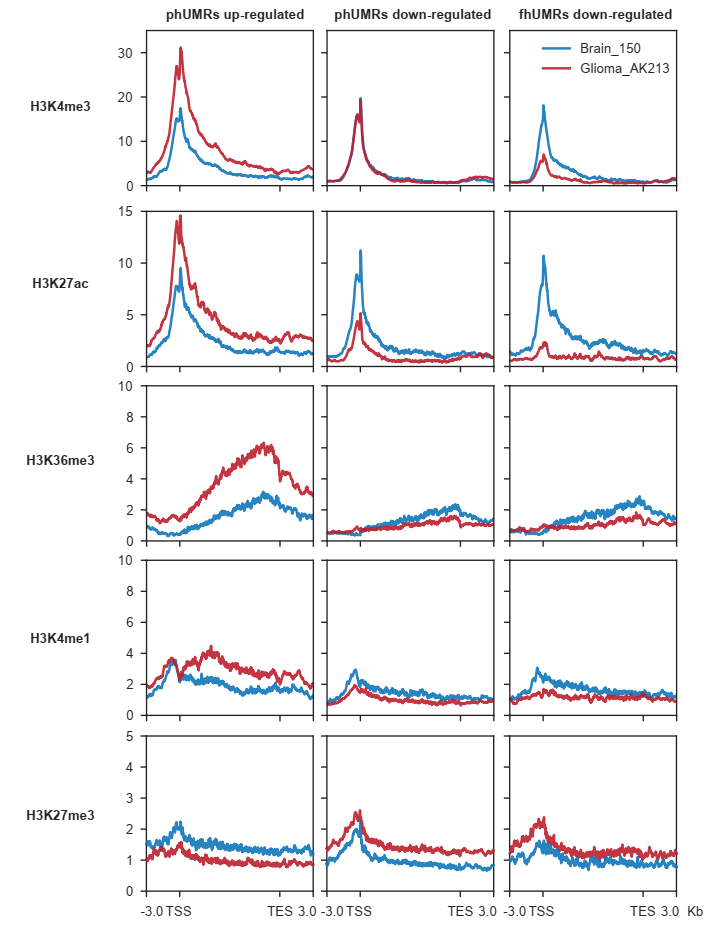


**Figure S8. Histone modification changes in differentially expressed genes within phUMR or fhUMR.** Average signals of histone modification mark around TSS and TES of differentially expressed genes between *IDH* mutant glioma (AK213, DKFZ) and normal brain tissue (150, Roadmap). The gene lengths were scaled into 9kb for phUMR and fhUMR related up/down-regulated genes.


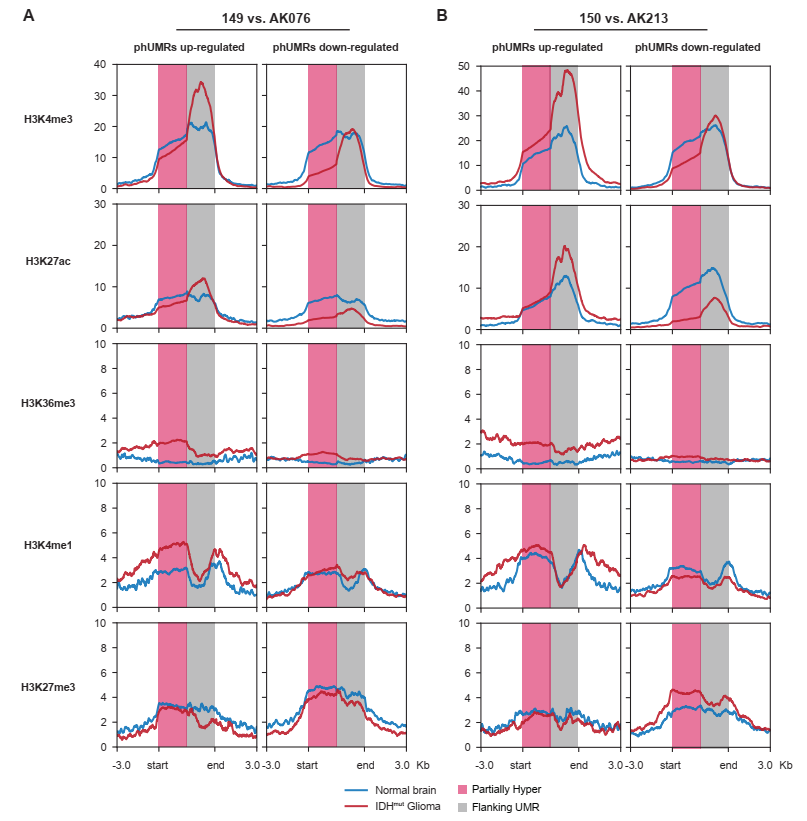


**Figure S9. Local histone modification changes on partial Hyper and flanking UMR in phUMRs.** A) Average signals of histone modification in up/down-regulated phUMRs among *IDH* mutant glioma (DKFZ, AK076) and normal brain tissue (Roadmap, 149). B) Average signals of histone modification in up/down-regulated phUMRs among *IDH* mutant glioma (DKFZ, AK213) and normal brain tissue (Roadmap, 150). Average ChIP-seq signals were scaled into 2kb for partial Hyper and flanking UMRs respectively.


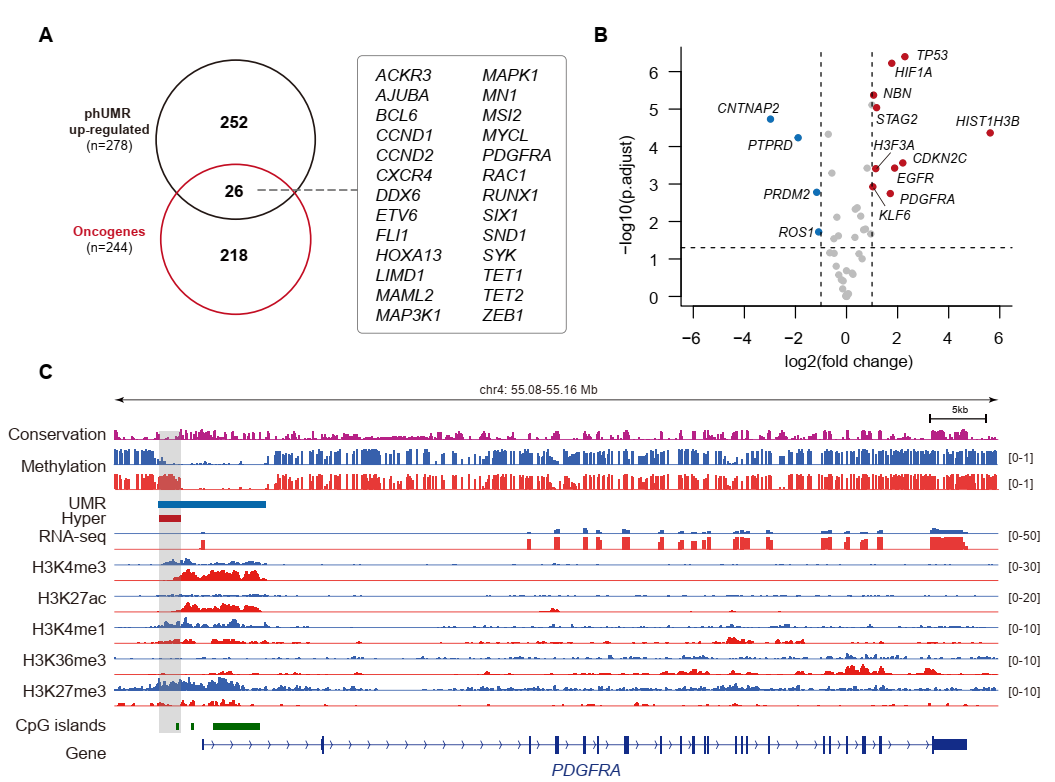


**Fig. S10. Partial methylation erosion on the promoter of glioma related genes.** A) The Venn diagram shows the overlap between phUMR up-regulated genes and oncogenes. B) Transcription patterns of 46 glioma related cancer genes. C) Genome Browser tracks depict DNA methylation, gene transcription and histone modification changes across *PDGFRA* in *IDH* mutant glioma (red) and normal brain tissue (blue).


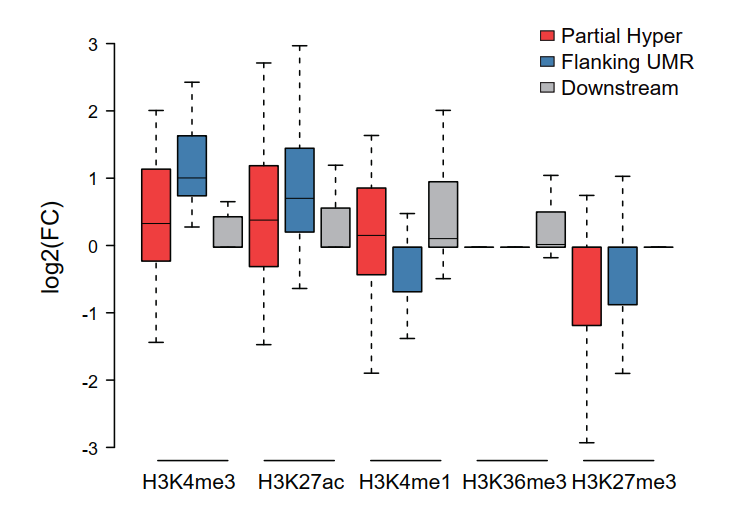


**Figure S11. Local changes of average histone modification signals at partial Hyper, flanking UMR and downstream regions of up-regulated oncogenes.** ChIP-seq signal fold changes were calculated among *IDH* mutant glioma (DKFZ, AK213) and normal brain tissue (Roadmap, 150). Partial Hyper and flanking UMR was determined by the border of hypermethylated CpGs in *IDH* mutant gliomas, and downstream represent the region from the border of phUMR to TES of oncogene.
